# Not on my team: Medial prefrontal cortex responses to ingroup fusion and unfair monetary divisions

**DOI:** 10.1002/brb3.1030

**Published:** 2018-06-22

**Authors:** Matthew A. J. Apps, Ryan McKay, Ruben T. Azevedo, Harvey Whitehouse, Manos Tsakiris

**Affiliations:** ^1^ Department of Psychology Royal Holloway University of London Egham UK; ^2^ Department of Experimental Psychology University of Oxford Oxford UK; ^3^ ARC Centre of Excellence in Cognition and its Disorders Macquarie University Sydney NSW Australia; ^4^ Department of Cognitive Science Macquarie University Sydney NSW Australia; ^5^ The Warburg Institute School of Advanced Study University of London London UK; ^6^ Institute of Cognitive and Evolutionary Anthropology University of Oxford Oxford UK

**Keywords:** fairness, fusion, group identity, prefrontal cortex, ultimatum game

## Abstract

**Objective:**

People are highly attuned to fairness, with people willingly suffering personal costs to prevent others benefitting from unfair acts. Are fairness judgments influenced by group alignments? A new theory posits that we favor ingroups and denigrate members of rival outgroups when our personal identity is fused to a group. Although the mPFC has been separately implicated in group membership and fairness processing, it is unclear whether group alignments affect medial prefrontal cortex (mPFC) activity in response to fairness. Here, we examine the contribution of different regions of the mPFC to processing from ingroup and outgroup members and test whether its response differs depending on how fused we are to an ingroup.

**Methods:**

Subjects performed rounds of the Ultimatum Game, being offered fair or unfair divisions of money from supporters of the same soccer team (ingroup), the fiercest rival (outgroup) or neutral individuals whilst undergoing functional Magnetic Resonance Imaging (fMRI).

**Results:**

Strikingly, people willingly suffered personal costs to prevent outgroup members benefitting from both unfair and fair offers. Activity across dorsal and ventral (VMPFC) portions of the mPFC reflected an interaction between fairness and group membership. VMPFC activity in particular was consistent with it coding one's fusion to a group, with the fairness by group membership interaction correlating with the extent that the responder's identity was fused to the ingroup.

**Conclusions:**

The influence of fusion on social behavior therefore seems to be linked to processing in the VMPFC.

## 1 INTRODUCTION

Humans are highly attuned to the fairness of others' actions (Fehr & Fischbacher, 2004; Pillutla & Murnighan, 1996; Rilling, King‐Casas, & Sanfey, 2008). People will willingly suffer a personal cost in order to prevent others earning an unfair division of rewards or resources. Such reactions to unfairness develop early, with children as young as eighteen months old reacting negatively to unequal distributions of rewarding stimuli (Sloane, Baillargeon, & Premack, 2012). However, our reactions to fair and unfair behaviors are influenced by the social identities of parties to an interaction (Bernhard, Fischbacher, & Fehr, 2006), but whether the effects of social identity on second‐party punishment are magnified by salient group alignments is less well‐understood.

Researchers have considered many distinct forms of alignment with groups (Swann, Jetten, Gómez, Whitehouse, & Bastian, 2012). An especially potent mode of group affiliation, highlighted in the recent literature, is identity “fusion” – in which the boundary between group and self is porous, and the individual experiences a “visceral feeling of ‘oneness’ with the group” (Swann & Buhrmester, 2015). Recent work has demonstrated that identity fusion is a powerful motivator of personally costly, progroup behaviors (Whitehouse, McQuinn, Buhrmester, & Swann, 2014; Whitehouse et al., 2017). For example, strongly fused individuals report more willingness to fight and die for their groups (Swann et al., 2014); are especially inclined to endorse sacrificing their lives for fellow in‐group members (but not out‐group members) in trolley dilemma scenarios (Swann, Gomez, Huici, Morales, & Hixon, 2010); and are especially likely to donate personal funds to support group members in difficulty (Buhrmester, Fraser, Lanman, Whitehouse, & Swann, 2015; Swann, Gomez, Dovidio, Hart, & Jetten, 2010). However, how the neural correlates of the fairness of another's actions are modulated by group membership, and how such a modulation may depend on the degree to which one is fused to one's group are poorly understood.

Research across species has highlighted three subregions of the medial prefrontal cortex (mPFC) that each play an important role in processing social information (Amodio & Frith, 2006); the ventromedial portions of the PFC (VMPFC; area 32), dorso‐medial PFC (DMPFC; areas 8 and 9 on the medial surface of the superior frontal gyrus), and portions of the anterior cingulate cortex (ACC; particularly areas 24a/b lying in the gyrus) (Apps, Lesage, & Ramnani, 2015; Apps, Rushworth, & Chang, 2016; Cooper, Kreps, Wiebe, Pirkl, & Knutson, 2010; Frith & Frith, 2006; Hare, Camerer, Knoepfle, & Rangel, 2010; Haroush & Williams, 2015; Lee, 2008; Lee & Seo, 2016; Lockwood, Apps, Roiser, & Viding, 2015). Lesions to these regions impair and reduce the execution of social behaviors and can lead to the inability to adhere to group or social norms (Anderson, Bechara, Damasio, Tranel, & Damasio, 1999; Gu et al., 2015; Hadland, Rushworth, Gaffan, & Passingham, 2003; Rudebeck, Buckley, Walton, & Rushworth, 2006; Tranel, Bechara, & Denburg, 2002). Notably, accounts have highlighted that these regions play important roles in processing information about group membership and categorization, and information regarding whether the actions of others violate group norms (Cikara, Jenkins, Dufour, & Saxe, 2014; Cikara & Van Bavel, 2014; Hein, Silani, Preuschoff, Batson, & Singer, 2010; Molenberghs, Gapp, Wang, Louis, & Decety, 2016; Molenberghs & Morrison, 2014).

Recently it has been suggested that neuroeconomic approaches may provide a powerful framework for understanding the neurobiological basis of altruistic and group behaviors (Everett, Faber, Crockett, & De Dreu, 2015). Studies using such approaches also implicate the mPFC in processing the fairness of other individuals, including when interacting during economic games such as the ultimatum game (UG) (Feng, Luo, & Krueger, 2015; Gabay, Radua, Kempton, & Mehta, 2014; Güth, Schmittberger, & Schwarze, 1982; Koenigs & Tranel, 2007; Sanfey, Rilling, Aronson, Nystrom, & Cohen, 2003). In the UG there are two players – the ‘proposer’ who makes an offer of how to split a pot of money with a ‘responder’. When the proposer offers to split the money equally the offer is considered ‘fair’ but when the proposed split is unequal – with more money going to the proposer than the responder – it is considered unfair. The responder must make a choice of whether to accept the offer, in which case the money is distributed as proposed; or to reject the offer, in which case neither player will receive any money. By manipulating offers received by the responder the UG offers an elegant way of examining how the brain responds to the fairness of others' actions (Sanfey et al., 2003). Recent meta‐analyses of neuroimaging studies of the UG have highlighted that the DMPFC, VMPFC, and ACC are influenced by the fairness of the proposal. However, crucially, none have examined how the VMPFC, DMPFC, and ACC respond to unfair offers from members of one's own or another social group. Is the response to unfairness in these regions modulated for ingroup members and by the degree of fusion to a group? Moreover, given that these regions have distinct functional and anatomical profiles in other domains of behavior (Barbas, Ghashghaei, Dombrowski, & Rempel‐Clower, 1999; Carmichael & Price, 1995; Nicolle et al., 2012), do they make distinct contributions to the processing of fairness in a group context?

**Figure 1:**
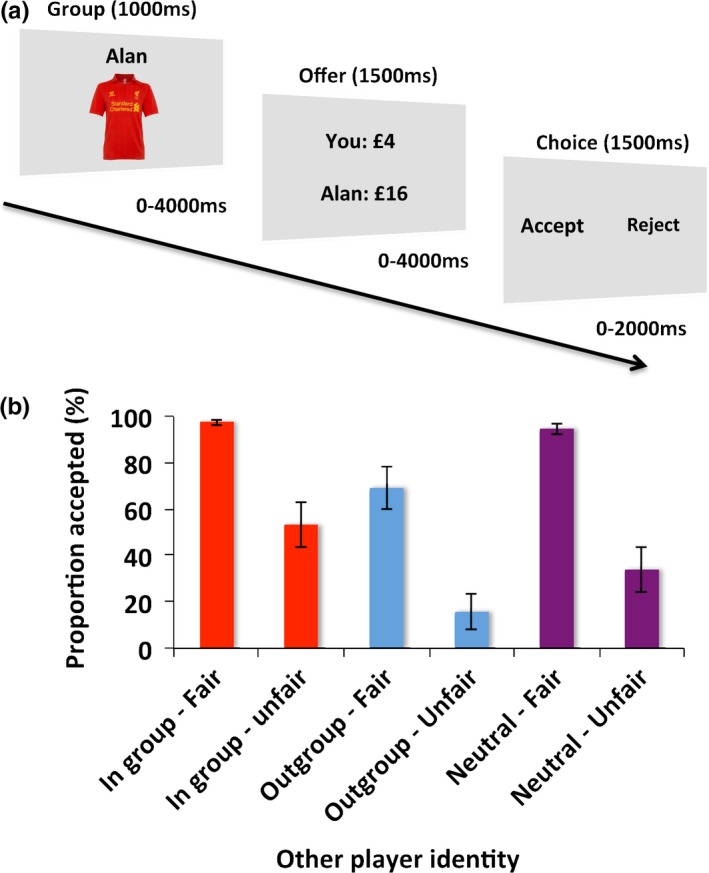
(a) Trial structure. Subjects played 180 one‐shot rounds of the UG as the ‘responder’ with a different ‘proposer’ on each trial. They received only the first name (‘Alan’) and what football team the proposer supported – indicated by the football shirt. The football shirt could be either that of the team supported by the participant (ingroup), that of the participant's supported team's biggest rival (outgroup), or no information about which team was supported was provided (neutral). Following a variable, uniformly distributed jitter they were then presented with the offer from the proposer. This could be either fair (£8 each) or unfair (as shown). Subjects were required to decide whether they would accept this offer and each player take the split of money or reject the offer and neither player receive any payment. After uniformly distributed variable jitter subjects were required to indicate their decision by pressing one of two keys on a keypad corresponding to the left and right hand side of the screen. The position of accept and reject randomly changed on every trial ensuring that activity at the time of the offers could not be related to motor preparation. (b) Behavioral results. Subjects accepted fewer unfair than fair offers, overall. However, there was also an effect of group membership. Subjects were more likely to reject offers from a fan of their biggest rival (outgroup‐blue) than from the ingroup (red) or neutral players (purple)

Here, subjects played 180 one‐shot rounds of the UG as the responder (Figure 1a), with members of either an ingroup, an outgroup, or neutral individuals ‐ whom subjects were given no information about – acting as proposers. To manipulate specifically group membership, subjects were given only one piece of information about each proposer, aside from their name – the football (soccer) team that they supported: Either the same team as them (ingroup), their team's biggest rival (outgroup), or someone whose team affiliation was unknown (neutral). This design allowed us to examine whether mPFC responses to fairness during the UG are modulated by the group membership of the person with whom they are interacting. This allowed us to test the hypothesis that portions of the mPFC may respond differently to unfair offers depending on the group membership of the proposers, and that this response may depend on how fused a subject is to their favored football team. Using this approach we dissect out the responses in the mPFC to the effects of group membership on the processing unequal divisions of money in the UG.

## 2 MATERIALS AND METHODS

### 2.1 Subjects

Subjects were originally 21, healthy right‐handed participants screened for neurological disorders (aged between 18 and 36; 6 female). Four subjects were excluded from the analyses for either failing to complete all of the trials, excessive head movements (multiple >3 mm inter‐slice movements) or being aware of the deception (see deception below) leaving a final sample of 17. All participants gave written informed consent. The studies were approved by the Royal Holloway, University of London Psychology Department Ethics Committee and conformed to the regulations set out in the CUBIC MRI rules of Operations. The subjects believed that they would be paid for their participation based on their decisions during the experiment (see below).

### 2.2 Apparatus

Subjects lay supine in an MRI scanner (3T Siemens Trio, CUBIC, Royal Holloway, University of London) with the fingers of the right hand positioned on an MRI‐compatible response box. Stimuli were projected onto a screen behind the subject and viewed in a mirror positioned above the subjects face. Presentation software (Neurobehavioral Systems, Inc., USA) was used for experimental control (stimulus presentation and response collection). A custom‐built parallel port interface connected to the Presentation PC received transistor‐transistor logic (TTL) pulse inputs from the response keypad. It also received TTL pulses from the MRI scanner at the onset of each volume acquisition, allowing events in the experiment to become precisely synchronized with the onset of each scan. Decisions were calculated off‐line, and event timings were prepared for subsequent general linear model (GLM) analysis of fMRI data (see event definition and modeling below).

### 2.3 Experimental design

In this study, we aimed to examine the contribution of the mPFC to the processing of fairness when interacting with ingroup compared to outgroup members and whether the degree of fusion to an ingroup member modulated mPFC response. To examine the response to fairness, subjects performed 180 rounds of the UG acting as the responder, to 180 different proposer's offers. Subjects received fair offers on half of the trials and unfair offers on the remainder. Thus, we could examine the effects of the fairness of offers on the BOLD response. In order to examine the effects of both group membership and fairness on the BOLD response subjects played each round of the UG with either an ingroup member, an outgroup member or a neutral individual. To manipulate group membership, subjects played each round of the UG with either someone who supported the same football team as them, someone who supported their team's biggest rival or a neutral individual. This 2 × 3 design allowed us to look for the effects of fairness and group membership on neural responses during the UG.

#### 2.3.1 Trial structure

Each trial began with subjects being informed of the first name of the proposer and being shown a football shirt i.e., a cue indicating the social group of the proposer. This shirt indicated the football team supported by the proposer. This was either the same team as them (ingroup), the team identified as the biggest rival (outgroup), or an individual where participants were provided no information about the team that was supported (neutral). Following this, the proposed “offer” of how to split the £16 was presented on the screen. This could either be an equal (fair) split or unequal (£12 to the proposer, £4 to the responder inside the scanner). Following this, subjects were presented with a screen where “accept” and “reject” were presented on the left and right‐hand side of the screen. To select a response, subjects pressed one of two buttons on a keypad, with each button corresponding to one side of the screen. The position of the accept and reject options varied randomly on each trial. This ensured that any activity time‐locked to the proposer's offer could not be related to the preparation of an action by the responder. Importantly, we jittered the group, offer and response cues independently in this study, allowing us to examine activity specifically time‐locked to fair and unfair offers independently from any other trial elements.

Note that due to the number of conditions in this experiment, it was not possible to use a range of offers to disentangle the magnitude of payoffs for self and other, from the overall fairness of offers. It is therefore possible that activity we observe may not be driven by fairness per se, but by the value of the payoffs for self and/or others. However, as the offered values were arising from either fair or unfair divisions of money any differential computation of value for self and other must be important for determining the fairness of the offers and thus how to behave in response to fair or unfair offers.

#### 2.3.2 Procedure

Participants were recruited from a large‐scale questionnaire project examining football fan attitudes. Participants had therefore all first completed a fusion questionnaire, a pictorial fusion scale [see (Gomez et al., 2011; Swann, Gomez, Seyle, Morales, & Huici, 2009) for details], and also a social identity questionnaire (Mael & Ashforth, 1992) with reference to how affiliated they were with their own team. The pictorial fusion method has proven powerful for identifying behavioral correlates of fusion, and how it can be distinguished from other types of group identity (Buhrmester et al., 2015; Gomez et al., 2011; Swann et al., 2009). In addition, participants were asked details about how strongly they supported their favorite football team. This included years supported and whether they ever went to watch their team play. The participants contacted to participate in the study were those who had supported the team for >5 years and who identified a sensible rival team. We also recruited so as to sample a range of fusion scores in terms of how strongly fused the participants were on the pictorial fusion scale, from the >3 upwards (note that one participant scored 4 on the initial questionnaire, but scored 3 on the day of scanning). This enabled us to examine whether people who felt their own team was an ingroup would show different behavioral or neural patterns to fair or unfair divisions of money during the UG.

Prior to scanning, participants were presented with a backstory about the nature of the proposers during the UG trials that they would be playing. Subjects were informed that this experiment was a part of a large‐scale project that had already been completed examining the behavior and personality traits of football fans. They were told that the offer they would see on a given trial would be from one person who had completed a set of questionnaires and one round of the UG in the role of proposer. We told subjects that we had selected a set of responses from people who supported the same team as them, from people identified as supporters of their biggest rival and from a group of people who we would not tell them anything about. Subjects were told these ‘neutral’ individuals could be fans of any team, or no team at all, but they would not receive any information about them, other than their name. This condition therefore provides the opportunity to examine individuals who are neither members of the ingroup nor members of an identified outgroup. Subjects were instructed that the other players were presented with the two splits of £16 (50/50 or 75/25) and that the other players were given no information about who their offers would be presented to. Importantly subjects were told that both their own payment and that of the other players could be influenced by the decisions they made on each trial.

Prior to the main scanning task, subjects performed a short practice of 12 trials (two trials of each of the six conditions). During the main experiment subjects played 180 trials of the UG, with 30 repetitions of each of the six conditions in a pseudo‐random order.

At the end of the experiment, participants were asked a series of standardized debriefing questions and were excluded if they reported an awareness or suspicion of the nature of the deception in the experiment (Apps et al., 2015). Subjects were also instructed not to tell any other individuals whom might take part in the experiment the nature of the deception of the task.

#### 2.3.3 Participant/outgroup selection and deception

To ensure that participants were responding to offers in the UG from true ingroup and outgroup individuals, participants completed a questionnaire about which football team they supported. Participants were asked how many years they had supported their favorite team and which team was their team's biggest rival. Participants were excluded if they had not supported their team for more than 5 years and also if they did not identify an appropriate rival (e.g., Everton's greatest rival would be Liverpool FC and not an unconnected team such as Southend United). For teams where there was more than one reasonable rival (e.g., Liverpool's biggest rivals could reasonably be Manchester United or Everton), the outgroup was the team the participants identified themselves as being the biggest rival to their supported team.

Participants were provided with several deceptive instructions prior to the experiments, which allowed us to ensure that subjects believed they were making real financial decisions that would influence their own payment and the payment of another individual. They were told that the offers they observed from proposers were taken from people who had completed one trial of the task as a part of a large online questionnaire project examining football fan behavior. They were told that the other people would be paid through an online system, based on the responses of subjects inside the MRI scanners. That is, if subjects chose to reject offers the other player would not be paid for their completion of the survey, whereas if they accepted an unfair offer the other person would be paid £12, and if they accepted a fair offer the other player would be paid £8. However, in reality, none of these elements of the project were true and participants were playing against a pseudorandom sequence of offers. Such an approach enabled us to maintain greater experimental control.

#### 2.3.4 Proposer names

Names were selected from a list of 100 male and 100 female names from a list of popular baby names from the year 2000 ( http://www.babycentre.co.uk/popular-baby-names). The order of names for the proposers was randomized across participants, however, equal numbers of names of each gender were presented in each condition to ensure that gender could not account for differences in rejection rates. In addition, three different pseudorandomly ordered sets of names were used across participants, preventing the possibility that names used could systematically bias neural or behavioral responses.

#### 2.3.5 Payment

Subjects were instructed prior to the task that they would receive payment for three of their decisions made during the main experiment, one for a response made to a fan of the same team as them, one for a rival and one for a neutral player. Three trials would be chosen by random selection from a hat at the end of the experiment and the decisions made on those trials would affect their own payment and the payment of the proposer on that round of the game. Importantly, subjects believed, therefore, that their decisions would influence their own payment for the experiment. In addition, we instructed subjects that their decisions would affect the other player, in that if they rejected the other player's offer the other player would not be remunerated for playing that round of the game (if selected for potential payment). Following completion of the experiment all subjects were in fact paid £30 for their participation.

### 2.4 Functional imaging and analysis

#### 2.4.1 Data acquisition

Scans were acquired on a Siemens Trio 3T scanner. T1‐weighted structural images were acquired at a resolution of 1 × 1 × 1 mm using an MPRAGE sequence. 910 EPI scans were acquired from each participant. 38 slices were acquired in an ascending manner, at an oblique angle (≈30˚) to the AC‐PC line to decrease the impact of susceptibility artifact in subgenual cortex (Deichmann, Gottfried, Hutton, & Turner, 2003). A voxel size of 3 × 3 × 3 mm (20% slice gap, 0.6 mm) was used; TR = 3s, TE = 32, flip angle = 85°. The functional sequence lasted 46 min. Immediately following the functional sequence, phase and magnitude maps were collected using a GRE field map sequence (TE1 = 5.19 ms, TE2 = 7.65 ms).

#### 2.4.2 Image preprocessing

Scans were preprocessed using SPM8 ( http://www.fil.ion.ucl.ac.uk/spm). The EPI images from each subject were corrected for distortions caused by susceptibility‐induced field inhomogeneities using the FieldMap toolbox. This approach corrects for both static distortions and changes in these distortions attributable to head motion (Hutton et al. 2002). The static distortions were calculated using the phase and magnitude field maps acquired after the EPI sequence. The EPI images were then realigned, and coregistered to the subject's own anatomical image. The structural image was processed using a unified segmentation procedure combining segmentation, bias correction, and spatial normalization to the MNI template (Ashburner & Friston, 2005); the same normalization parameters were then used to normalize the EPI images. Lastly, a Gaussian kernel of 8 mm FWHM was applied to spatially smooth the images in order to conform to the assumptions of the GLM implemented in SPM8. The timings and randomization of stimulus presentations were checked before conducting the study, to ensure that the resulting design was not adversely affected by rank deficiency. As a result we were able to analyse activity time‐locked to the offers without the confounding effects of subsequent responses.

#### 2.4.3 Event definition and modeling

To examine activity time‐locked to offers in the UG, we created a GLM with six regressors of interest (pertaining to the 2 (fairness) × 3 (group) design. In addition single regressors were included for the group cue at the start of each trial and also for the response cue. Although activity at the time of the response cue could theoretically be broken down by condition, as the choices made by subjects were so different by conditions, interpreting activity time‐locked to these cues could be entirely driven by motor preparation or decision‐related activity that is unrelated to group membership or fairness per se. Therefore, our analyses are predominently focused on activity time‐locked to the offers and not response cue. Regressors were constructed for each of these events by convolving the event timings with the canonical Haemodynamic Response Function (HRF). The effects of head motion were modeled in the analysis by including the six parameters of head motion acquired during preprocessing as covariates of no interest.

#### 2.4.4 Second‐Level analysis

Random effects analyses (Flexible‐Factorial ANOVA) were applied to determine voxels significantly different at the group level. SPM{t} images from all subjects at the first‐level were entered into second‐level flexible factorial design matrices. T‐contrasts and F‐contrasts were conducted in each of the GLMs.

#### 2.4.5 Contrasts and covariate analysis

Three main contrasts were conducted to test the hypothesis that portions of the mPFC would show an effect of fairness and group‐membership. The first was a fairness by group interaction averaging across both the outgroup and neutral conditions. The second and third pertained to fairness by group interactions either between ingroup and outgroup or between ingroup and neutral.

To examine the relationship between fusion and the response to fairness between groups we performed covariate analyses at the second level with the scores from the fusion pictorial score as covariates of the first contrast above. We performed this covariate analysis in three ways (i) performing a whole‐brain covariate analysis and examining the voxels which showed an effect that overlapped with those only showing an interaction effect and (ii) running the same analyses but performing small volume correction using masks of areas 24, 32, 8 and 9 (see below) and (iii) by averaging over all of the voxels in the masks and performing the covariate analysis. This allowed us to identify a cluster that showed a significant covariation but also ensured that an independently identified region of interest shows a significant covariation of fusion with the group by fairness interaction. We then performed 6 separate covariate analyses by conditions to examine whether the response in the mPFC to any particular condition varied with the degree of fusion to one's ingroup.

#### 2.4.6 Multiple comparison correction and anatomical specificity

To correct for multiple comparisons we used two approaches. Firstly, for the main, first contrast above we set a voxelwise threshold of *p* < 0.001 uncorrected, but then corrected at the cluster threshold of *p* < 0.05 FWE (Eklund, Nichols, & Knutsson, 2016; Woo, Krishnan, & Wager, 2014). To then specify the location of our results with greater precision, we used masks of areas 8, 9, 24 and both the dorsal and ventral portions of area 32 from the study of Neubert and colleagues (Neubert, Mars, Sallet, & Rushworth, 2015). These masks were derived from connectivity based parcellations of the frontal cortex, and are also consistent with cytoarchitectonic parcellations. As such, by using these masks we are able to localize activity to regions that are anatomically and functionally distinct. Masks for these regions were selected based on the meta‐analyses of Feng et al. (2015) and Gabay et al. (2014). The peak coordinates from these studies for signaling fairness lie within the masks of areas 8, 9, 32, and 24. All of the reported results were therefore significant using either a cluster or voxelwise approach to correcting for multiple comparisons.

## 3 RESULTS

Subjects (*N* = 17) played as the responder in 180 rounds of the UG with either supporters of the same football team as the subject (ingroup), supporters of the team identified by the subject as their team's biggest rival (outgroup), or an individual who could be a supporter of any team or not a football fan at all (neutral).

### 3.1 Ingroup favoritism in football fans

Economic theories state that self‐interest maximizers should always accept nonzero offers in the UG. However, many studies have shown that people are willing to reject unequal (unfair) divisions of money (Koenigs & Tranel, 2007; Pillutla & Murnighan, 1996). Using our design we were able to examine the influence that fairness and group membership have on people's acceptance of offers in the UG (Figure 1b). A 2 × 3 repeated measures ANOVA on the acceptance rates ‐ arcsine transformed to ensure a normal distribution ‐ revealed a main effect of the fairness of the offer, a main effect of group membership of the proposer, but no interaction (Fairness: *F*(1,16) = 54.78, *p *<* *0.001; Group: *F*(1.67,26.6) = 14.74, *p *<* *0.001; Fairness × Group: *F*(1.78,29.49) = 0.89, *p *=* *0.41). These effects were driven by significantly greater acceptance of fair than unfair offers in all three groups (ingroup: t(16) = 5.73, *p *<* *0.001; outgroup: t(16) = 4.84, *p *<* *0.001; neutral: t(16) = 6.58, *p *<* *0.001) as well as significant differences in acceptance rates between ingroup and outgroup, the outgroup and neutral, and a marginal difference between ingroup and neutral (ingroup‐outgroup: t(16) = 4.43, *p *<* *0.001; neutral‐outgroup: t(16) = 3.93, *p *<* *0.001; ingroup‐neutral: t(16) = 2.07, *p* = 0.055). Thus, although subjects were less likely to accept unfair offers in all conditions, they were even less likely to accept offers from the outgroup than ingroup or neutral proposers. Moreover, the absence of a fairness by group membership interaction points to subjects rejecting more *fair* outgroup offers than fair ingroup or neutral offers. In other words, participants were willing to incur a personal cost to punish even well meaning outgroup members (Diekhof, Wittmer, & Reimers, 2014). No relationships between choices in the UG and the pictorial fusion measure were identified (*ps *> 0.05).

### 3.2 Imaging results

To examine whether the responses of the DMPFC, VMPFC, or ACC were driven by an interaction between fairness and group membership, we examined activity time‐locked to the offer cues. A neural response to fairness that is related specifically to processing information only about ingroup members should show a differential response to the fairness of offers from ingroup members, in comparison to the responses to the fairness of offers from both outgroup and neutral members. The response to the fairness of ingroup offers should therefore be (i) different to the average effect of the unfair‐fair offers from all noningroup members, and (ii) different separately to unfair‐fair offers compared to the neutral and outgroups. Although behaviorally there was evidence of a difference in the acceptance of offers from outgroup and neutral group members, it is plausible that activity in some regions may show a unique response to ingroups relative to all others. We therefore performed two sets of analyses to look for an ingroup unique response to fairness that is different from responses to outgroup and neutral individuals presented in the following two sections.

#### 3.2.1 An interaction between fairness and group membership in the mPFC

**Figure 2:**
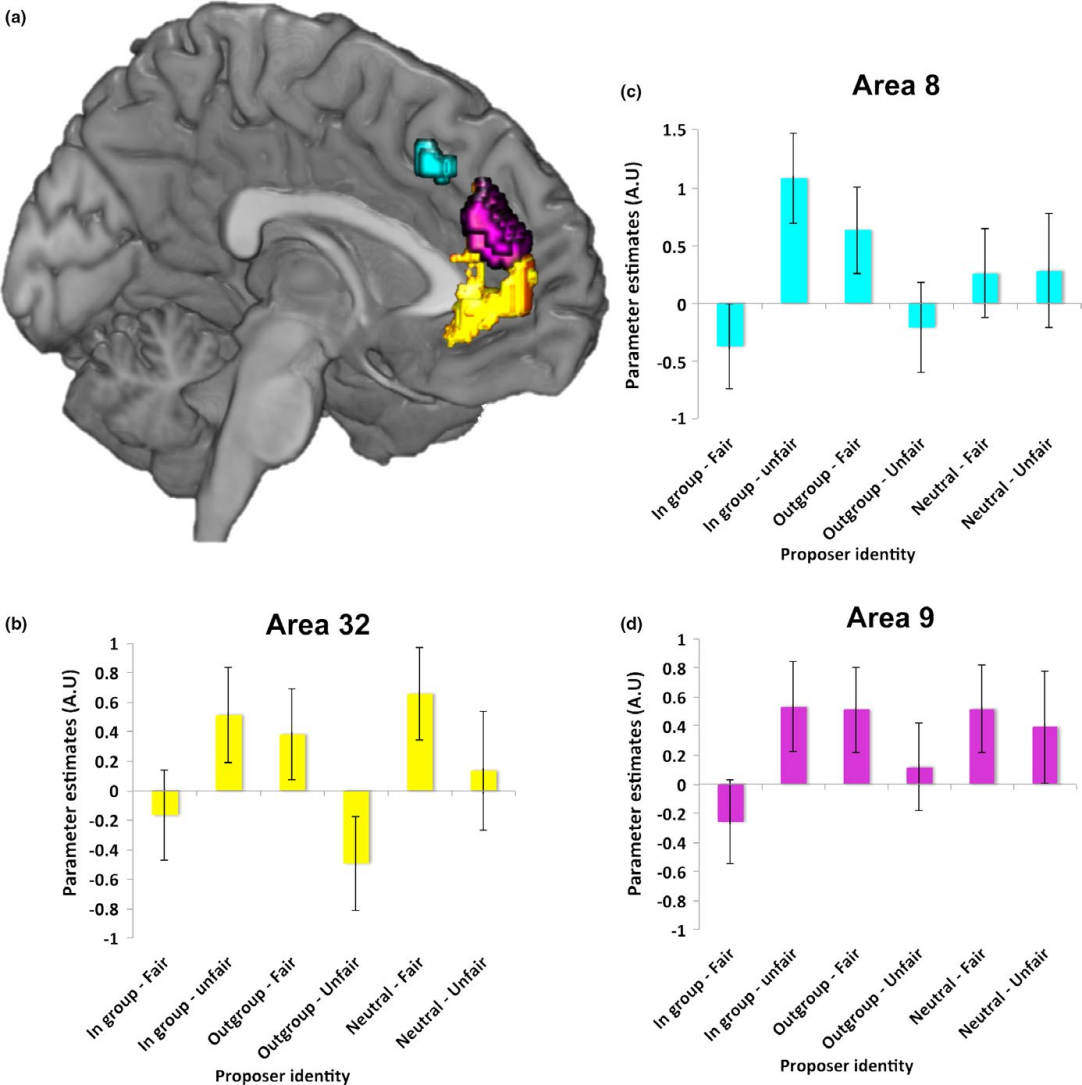
Activity in regions that showed a Fairness (unfair‐fair) × Group (ingroup –[outgroup and neutral] interaction) (*p* < 0.001 uncorrected for display purposes). Parameter estimates from the peak of each of these clusters is shown for the VMPFC (b), and for the two DMPFC regions (c and d). (b) Activity in the VMPFC region survived whole‐brain, cluster‐correction as well as a mask of area 32. Voxels in this region also showed a significant difference in the response to fairness between the ingroup and outgroup, as well as between the ingroup and neutral. This suggests the VMPFC may be crucial for processing information specifically about ingroups. Activity in the DMPFC only showed a difference in their response to fairness between the ingroup and the averaged response of ingroup and neutral (c and d). Error bars depict *SEM*

We performed a 2 × 2 F‐contrast between fairness (unfair‐fair) and group (ingroup – [outgroup + neutral]) to examine activity driven differentially by fairness that also differed between an ingroup player and any other individual (neutral or outgroup) in the mPFC. This contrast revealed a cluster that extended over a large portion of the mPFC (Figure 2), including both the DMPFC (areas 8 and 9) and VMPFC (area 32), in the same region as recently identified within a meta‐analysis of UG studies (Feng et al., 2015; Gabay et al., 2014). This large cluster survived whole brain cluster correction (*p < *0.001 uncorrected voxel‐wise; *p *<* *0.05 FWE cluster‐correction) at the level recommended in recent analyses of corrections for multiple comparisons (Eklund et al., 2016; Woo et al., 2014). Notably, we did not find any responses in other regions implicated in the UG, such as the Insula, showing a group by fairness interaction effect. However, we did find a cluster in the Isthmus (18, −44, 12; *Z* = 4.15, *p < *0.001 voxel‐wise; *p *<* *0.05 FWE cluster). Furthermore, clusters in these same regions were also present when performing the full 2 × 3 interaction between fairness (Fair – Unfair) and group (Ingroup X Outgroup X Neutral) at the same statistical threshold as all other reported results.

To examine with greater anatomical precision which regions of the mPFC this cluster encompassed, we also used masks of areas 8, 9, 32, and 24 which were defined from a resting‐state parcellation of the mPFC by Neubert and colleagues (Neubert et al., 2015). These masks allowed us to localize results with greater anatomical specificity. Using this approach, we found clusters within areas 8, 9, and 32 that survived small volume correction for the number of voxels present within each mask (area 8: 0, 28, 44; *Z* = 3.83, *p < *0.05 svc area 8 mask*;* area 9: 8, 44, 22; *Z* = 3.75 svc area 9 mask, *p < *0.05 svc*;* area 32: 8, 48, 4; *Z* = 4.16, *p < *0.05 svc mask). However, no voxels were present within the ACC gyrus area 24. Thus, both ventral and dorsal portions of the mPFC show an interaction between the how fairly money is being proposed to be divided, and the group membership of the proposer.

#### 3.2.2 Ingroup specific response in the VMPFC

To further examine whether the responses in the VMPFC and DMPFC truly reflected a differential signaling of fairness between ingroup and individuals from any other group we performed two additional contrasts. First, we looked for a fairness (unfair‐fair) by group interaction between only the ingroup and outgroup (ingroup‐outgroup) members. Second, we performed a fairness by group contrast but only for the ingroup and neutral proposers. A response to both of these contrasts would indicate that a region is signaling fairness to the ingroup differently than for both the outgroup and neutral. The first contrast revealed a large cluster extending over both the VMPFC and DMPFC (*p < *0.001 uncorrected voxel‐wise; *p *<* *0.05 FWE cluster‐correction) that overlapped considerably with the cluster that responded to the fairness x ingroup‐all other groups contrast. These clusters contained significant effects across areas 8, 9, and 32 (area 8: 0, 26, 44; *Z* = 4.75, *p < *0.01 FWE svc‐area8 mask*;* area 9: 6, 44, 22, *Z* = 4.49, *p < *0.01 FWE svc‐area9 mask; area 32: 2, 54, 0, *Z* = 4.24, *p < *0.05 FWE svc‐area32 mask). An additional cluster was also found in the Cuneus (4, −58, 42; *Z* = 4.21, *p < *0.001 voxel‐wise; *p *<* *0.05 FWE cluster). Thus, both the DMPFC and VMPFC show a differential response to fairness between the ingroup and the outgroup proposers.

However, the second contrast revealed no voxels in the DMPFC, but a significant effect in the VMPFC (area 32: 12, 56, 6, *Z* = 3.61, *p *<* *0.05 FWE svc *area32 mask*). No other region showed this profile. This highlights that the VMPFC responds differentially to fairness between ingroup and neutral, and between ingroup and outgroup, suggesting that the response may be important for distinguishing ingroups from outgroup and neutral individuals. In contrast, the DMPFC response to fairness appears to be present only when comparing ingroup to outgroup and not ingroup to neutral.

#### 3.2.3 Fusion to the ingroup modulates VMPFC response to fairness

**Figure 3:**
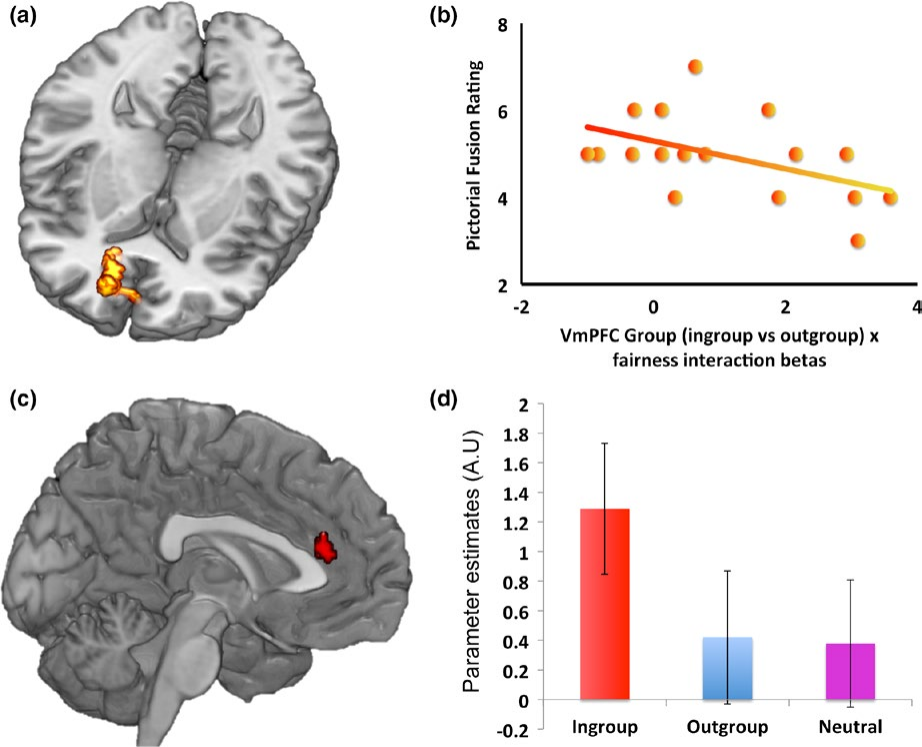
(a) Voxels in the VMPFC showed covariation between fusion to one's football team and the interaction between Fairness and Group (ingroup –[outgroup and neutral] interaction when seeing offers; *p* < 0.001 uncorrected for display purposes. Cluster survived small volume correction for area 32).(b) correlation between the VMPFC interaction response and football fusion scores (note that this graph is not a statistical representation of a correlation, but for display purposes only). The ACCg (c) showed a main effect of group during the response phase, driven by differences in the response of this region between ingroup and outgroup as well as ingroup and neutral as shown in the parameter estimates from the peak voxel (d)

To examine whether the degree to which an individual was fused to the team they supported modulated the responses of the mPFC to fairness, we examined whether the interaction between Fairness and Group (ingroup – [outgroup + neutral]) covaried with fusion ratings from the pictorial fusion scale using a covariate analysis. A cluster that extended over the VMPFC (Area 32) and portions of the pregenual ACC (area 24) had activity in which the interaction between group and fairness was modulated by the degree to which individuals were fused to the group (Figure 3). Many of these voxels overlapped with voxels that displayed the interaction effect (area 32: 10,42,14; *Z* = 3.61, *p* < 0.05 FWE‐svc area32 mask, corrected *p* = 0.009). No other region showed a significant effect. In addition, when averaging over all of the voxels in area 32 and covarying the fusion scores with the interaction effect we also found a significant effect (*r* ^*2*^ = 0.21, *p* = 0.02). Note that in this analysis the voxels are independently identified with respect to the interaction and covariate effects. Thus, fusion to a group modulates the interaction between fairness and group membership.

#### 3.2.4 Anterior cingulate gyrus signals ingroup response when indicating a choice

To examine whether any regions signaled information when subjects made a choice about whether to accept or reject the offer we performed analyses at the time of choice. As subjects accepted almost all fair offers from ingroup members and only a very small proportion of unfair outgroup offers, it was not possible to examine activity related to specific accept or reject decisions by condition. That is, there were too few samples for some subjects in order that any analyses could be performed to look at fairness by choice effects. However, we performed the same analysis of Group by Fairness that was performed on the offers. We found no regions signaling an interaction, even at a reduced threshold, nor did we find a main effect of fairness. However, we found a main effect of ingroup versus other groups (ingroup – [outgroup + neutral]) in the ACCg (0, 36, 18; *Z* = 3.9, *p* < 0.001 uncorrected voxelwise, *p* < 0.05 FWE clusterwise). This region (Figure 3) showed a significant difference between ingroup and outgroup (0, 36, 18; *Z* = 3.9, *p* < 0.001 uncorrected voxelwise, *p* < 0.05 FWE clusterwise) and also separately an overlapping cluster was also significant at a slightly reduced threshold between ingroup and neutral (0, 36, 16; *Z* = 3.48, *p* < 0.005 uncorrected voxelwise, *p* < 0.05 FWE clusterwise). Notably all of these results also survived correction when using a mask of the ACCg as a voxelwise small volume correction (Neubert et al., 2015). The ACCg is therefore sensitive to the group membership of an individual when making choices based on the fairness of their actions. No other region showed any main effects or interactions even at a reduced threshold (*p *<* *0.005 uncorrected).

## 4 DISCUSSION

Social interactions are shaped by our alignment with groups. In this study, we examined how the intensity of felt connection to a football team influences how the brain responds to divisions of money that are (unfair) unequal when the offer is from a supporter of the same team (ingroup member), a ‘rival’ supporter (outgroup member) or a neutral individual. Our results show that people will reject both equal and unequal offers from someone who supports a rival team more readily than from a supporter of the same team as them, or from a neutral individual. fMRI results point to the mPFC – a region previously implicated in research separately examining responses to fairness and group membership ‐ as showing an interaction between these factors. Our results suggest that the mPFC and particularly its more ventral portions, respond differently to divisions of money depending on the group membership of an interaction partner, and that this response is modulated by the intensity of one's affiliation (fusion) to a social group.

Our finding that participants were more likely to reject offers – whether unfair or fair – from outgroup members than from ingroup members or neutral individuals, adds to a growing literature on “parochial altruism”, in which people undertake personally costly acts to benefit their groups by harming other groups. These results also support a growing body of evidence that people are more likely to prevent outgroup members obtaining monetary rewards, relative to ingroup members. People are more likely to reject offers in the UG from outgroups, and the rejection of UG offers increases with increasing social distance to the outgroup (De Dreu et al., 2010; Everett et al., 2015; Kubota, Li, Bar‐David, Banaji, & Phelps, 2013; Mendoza, Lane, & Amodio, 2014; Reimers & Diekhof, 2015; Yamagishi & Mifune, 2016)**.** Behaviorally our results support such findings, underscoring the powerful effects that group membership has on the willingness to be altruistic toward others.

Previous neuroimaging studies have separately highlighted that mPFC activity is sensitive to group membership and the fairness of others. Recent meta‐analyses have highlighted that the DMPFC and the Insula are key regions that respond differentially to equitable and inequitable offers in the UG (Feng et al., 2015; Gabay et al., 2014). Similarly, a large number of different paradigms have shown that several regions including both the DMPFC and VMPFC respond to ingroup and outgroup members differently (Cikara & Van Bavel, 2014; Cikara et al., 2014; Harris & Fiske, 2006; Hein et al., 2010; Molenberghs et al., 2016). The response in these regions has been shown to predict helping of outgroup members and differential punishment of ingroup versus outgroup behaviors. Moreover, lesions to these regions are associated with changes in social cognition abilities and social behaviors (Barrash, Tranel, & Anderson, 2000; Bechara, Tranel, & Damasio, 2000; Blair & Cipolotti, 2000; Gu et al., 2015; Hadland et al., 2003; Saver & Damasio, 1991; Tranel et al., 2002). Here, we show that three key regions of the mPFC (putatively areas 8, 9 and 32) show differential responses to (un)fairness depending on whether the individual performing the behavior is an ingroup, outgroup, or neutral individual. This points to large portions of the mPFC being sensitive to the effects of group membership on how the behaviors of others are processed. However, by having participants play the UG with neutral proposers as well as with ingroup and outgroup individuals, we were able to show that the response of the VMPFC in particular fits a profile of signaling information about ingroup members differently from all other people. This finding therefore adds to recent evidence that suggests that the VMPFC may play an important role in in‐group favoritism.

Intriguingly, previous studies have identified that activity in the regions of the DMPFC and VMPFC is increased in response to unfair offers relative to fair offers (Feng et al., 2015; Gabay et al., 2014). Surprisingly, we identified this pattern of activity in these regions but only for offers from ingroup members, not for outgroup or neutral individuals. This suggests that the response of the MPFC in classical UG experiments may mirror what happens only when interacting with ingroup members. Such a finding raises questions about how the MPFC response to fairness may depend on the social context or on the individual with whom one is interacting. Although it is not possible to determine this from the present experiment, our findings raise two possibilities: one is that invoking group membership decreases the salience of the offers from noningroup members, and thus inhibits the signaling of fairness in these regions. Second is that in classical experiments contact with the other players ‐ or even simply knowledge that other individuals are part of the experiment ‐ forms a minimal group (Hewstone, Rubin, & Willis, 2002) and leads to the observed pattern of activity. Future work will need to test such possibilities behaviorally and examine whether they can be used to better understand MPFC responses in the UG.

Crucially, the notion of favoring one's ingroup is a key component of how “fused” people are to their social group, with people who are fused exhibiting more prosocial behaviors toward ingroup members (Swann & Buhrmester, 2015; Swann, Gomez, Dovidio, et al., 2010; Swann, Gomez, Huici, et al., 2010; Swann et al., 2014). However, the mechanisms in the brain that might mediate the influence of our affiliation to a group on our responses to the behavior of another person, have not previously been explored. Notably, the effects are in a similar region to those identified in studies examining self‐related processing, including self‐reflection and studies showing effects of similarity on neural responses when people made judgments about other people (Ames, Jenkins, Banaji, & Mitchell, 2008; Cikara et al., 2014; Jenkins & Mitchell, 2011; Kelley et al., 2002; Mitchell, Macrae, & Banaji, 2006; Moran, Macrae, Heatherton, Wyland, & Kelley, 2006) and also depend on the degree to which individuals shift their behaviors to conform with others (Apps & Ramnani, 2017). This raises the possibility that activity in response to others in this region may become more similar to self when interacting with ingroup members, and that this mergence is greater when an individual is highly fused to the group. Indeed, recent work has suggested that representation of self and other during reward‐related interactions may merge within areas 32 and 9 in the MPFC, particularly overlapping with the regions that were differentially engaged by fusion to a group in this study (Wittmann et al., 2016). Here, rather than mergence depending on the actions of the other person, we show that VMPFC responses are also influenced by one's fusion to a social group**.**

Recent meta‐analyses of neuroimaging studies of fairness have highlighted the mPFC as a key region in which activity differs on receipt of fair vs. unfair offers in the UG (Feng et al., 2015; Gabay et al., 2014). However, our results suggest that the response in the mPFC may not be simply to whether another person is proposing a fair or unfair division of resources. Specifically, we show that the response of the DMPFC and VMPFC to (un)fairness depends on whether the person we are interacting with is a member of our ingroup, or an outgroup or neutral individual. This raises the possibility that the processing of (un)fairness is influenced by expectations or beliefs about others given their group membership. This follows decades of research showing that people have favorable in‐group attitudes and unfavorable out‐group attitudes, even when group categorizations are only minimally emphasized (Hewstone et al., 2002). Our study extends these findings by showing that these biases can have a significant effect on the response of the mPFC to others’ behaviors (Aoki et al., 2014; Cikara & Van Bavel, 2014; Izuma, 2013), and moreover, in the VMPFC this response is dependent on the degree to which the individual is *fused* to the ingroup.

Is activity in the VmPFC related to processing value or to fairness? The portion of the VMPFC identified in this study has been consistently linked to the processing of the value of rewards for ourselves and also others (Rushworth & Behrens, 2008; Smith, Clithero, Boltuck, & Huettel, 2014). Both neurophysiological recordings in monkeys and neuroimaging studies in humans show that neurons in this region predict the value of rewards that we – and others – will receive (Apps et al., 2016; Garvert, Moutoussis, Kurth‐Nelson, Behrens, & Dolan, 2015; Hill, Boorman, & Fried, 2016; Nicolle et al., 2012). It is therefore possible that activity in this study could be related to the differential payoffs to self and other, rather than to fairness per se. In this study, such a possibility was not directly controlled for. However, there is evidence that the VMPFC and DMPFC regions differentially code between fair and unfair offers, including in studies where the magnitudes of offers to self and other were controlled for [see (Feng et al., 2015) for a meta‐analysis]. Studies that have directly compared activity to the same magnitude of rewards being delivered to self and other, find that activity in the VMPFC and DMPFC is not different between self and other rewards (Apps & Ramnani, 2014; Lockwood et al., 2015; Yoshida, Saito, Iriki, & Isoda, 2012). If activity in these regions is equally sensitive to payoffs to self and other, but is different to when these monetary values are unfairly divided, it suggests that the response in these regions is sensitive to the fairness of offers. Here, differences are identified between equal and unequal divisions of money. This would provide tentative evidence to support the notion that activity in the DMPFC and VMPFC may be modulated by the interaction between fairness and group membership. However, future work should directly test whether this activity is explicitly related to the fairness of the offer.

Another possibility is that the intrinsic rewarding value of punishing another is traded off against the monetary value, such that it becomes more rewarding to punish an outgroup member for the same sum of money (Rai, Valdesolo, & Graham, 2017). Such an account could plausibly explain the behavioral results in this study – the increased rejection of all offers from outgroup members. However, it cannot explain activity in the VMPFC, as there was no difference in activity between outgroup and neutral between unfair and fair offers, even though behaviorally people rejected more from outgroup than neutral.

Recently, several neuroimaging and neurophysiological studies have shown that the ACCg also plays a crucial role in processing the value of rewards that another will receive as opposed to rewards we will receive ourselves (Apps et al., 2016). This includes evidence that the ACCg is sensitive to the predicted value of a reward another will receive, and also to the decisions others make about rewards, as well as the offers others make to us during the UG (Apps & Ramnani, 2014; Apps et al., 2015; Gabay et al., 2014; Lockwood et al., 2015). Whilst the design of this experiment was not optimized to look at how the brain processes the rewards received by others, the ACCg did respond differently to ingroup individuals compared to others. Notably, this is a distinct region of the ACC from that engaged when processing the similarity of others (Cikara & Van Bavel, 2014; Losin, Cross, Iacoboni, & Dapretto, 2014). This suggests that the response of ACCg to rewards that others will receive may depend on their group membership. Moreover, the connections between the ACCg and the VMPFC (Apps et al., 2016; Balsters, Mantini, Apps, Eickhoff, & Wenderoth, 2016; Vogt, 2009) and the fact that both regions showed a profile indicative of signaling information in an ingroup centered reference frame suggests that they may form part of a network that plays an important role when interacting in social groups (Balsters et al., 2017; Sallet et al., 2011).

It is important to note that this study is to a degree limited by a relatively small sample size. Although the reliability of the results is increased by being designed in a manner that increases power within subject by using a large number of repetitions of each cell of the design, and by only focusing on statistically robust results in hypothesized regions, future work will need to replicate these effects and test them in larger samples.

The nature and intensity of group affiliations can have a powerful influence on our behavior. Here, we show that it also can have significant influences on information processing in the mPFC. In particular, we show that across the mPFC the sensitivity of these regions to the fairness of another's actions depends on whether they support the same football team. However, the response in the VMPFC also varied with how fused an individual was to the football team they supported and showed a profile suggesting an ingroup prioritizing response to fairness. These findings point to the important role that group memberships and fairness can have on social behavior and the response of the mPFC.
